# Dibromido[methyl 2-(quinolin-8-yl­oxy-κ^2^ *N*,*O*)acetic acid-κ*O*]mercury(II)

**DOI:** 10.1107/S1600536812028085

**Published:** 2012-06-23

**Authors:** Rui-Feng Song, Xue-Hua Zhu, Yu-Hong Wang

**Affiliations:** a School of Chemistry and Bioengineering, Suzhou University of Science and Technology, Suzhou 215009, People’s Republic of China

## Abstract

In the title complex, [HgBr_2_(C_12_H_11_NO_3_)], the Hg^II^ ion has a distorted core trigonal–planar geometry comprising two Br atoms and one quinoline N atom of the methyl 2-(quinolin-8-yl­oxy)acetic acid ligand. The angles around the Hg atom vary from 100.31 (15) to 152.65 (4)°. Two additional Hg⋯O inter­actions [2.739 (1) and 2.905 (1) Å] complete the coordination sphere about the Hg^II^ atom.

## Related literature

For quinoline derivatives, see: Ghedini *et al.* (2002 ▶); Inomata *et al.* (1999 ▶); Jotterand *et al.* (2001 ▶). For transition metal coord­ination compounds with 8-quinolinyloxyacetic acid and its derivatives as ligands, see: Cheng *et al.* (2007 ▶); Song *et al.* (2004 ▶); Wang, Song *et al.* (2005 ▶); Wang, Fan *et al.* (2008 ▶).
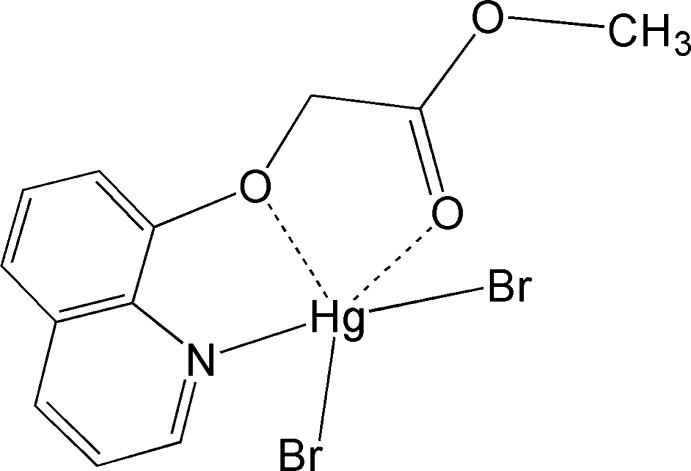


## Experimental

### 

#### Crystal data

[HgBr_2_(C_12_H_11_NO_3_)]
*M* *_r_* = 577.63
Triclinic, 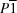

*a* = 7.3132 (8) Å
*b* = 9.9385 (10) Å
*c* = 10.9902 (10) Å
α = 72.102 (11)°
β = 74.966 (12)°
γ = 70.740 (11)°
*V* = 706.40 (14) Å^3^
*Z* = 2
Mo *K*α radiation
μ = 16.55 mm^−1^
*T* = 223 K
0.50 × 0.40 × 0.20 mm

#### Data collection

Rigaku Saturn diffractometer
Absorption correction: multi-scan (*REQAB*; Jacobson, 1998 ▶) *T*_min_ = 0.044, *T*_max_ = 0.137
6021 measured reflections
2599 independent reflections
1949 reflections with *I* > 2σ(*I*)
*R*_int_ = 0.068

#### Refinement

*R*[*F* ^2^ > 2σ(*F* ^2^)] = 0.038
*wR*(*F* ^2^) = 0.074
*S* = 0.80
2599 reflections
174 parameters
H-atom parameters constrained
Δρ_max_ = 2.41 e Å^−3^
Δρ_min_ = −2.04 e Å^−3^

### 

Data collection: *CrystalClear* (Rigaku, 2001 ▶); cell refinement: *CrystalClear*; data reduction: *CrystalStructure* (Rigaku, 2004 ▶); program(s) used to solve structure: *SHELXS97* (Sheldrick, 2008 ▶); program(s) used to refine structure: *SHELXL97* (Sheldrick, 2008 ▶); molecular graphics: *SHELXTL* (Sheldrick, 2008 ▶); software used to prepare material for publication: *SHELXTL*.

## Supplementary Material

Crystal structure: contains datablock(s) I, global. DOI: 10.1107/S1600536812028085/gg2082sup1.cif

Structure factors: contains datablock(s) I. DOI: 10.1107/S1600536812028085/gg2082Isup2.hkl

Additional supplementary materials:  crystallographic information; 3D view; checkCIF report

## Figures and Tables

**Table 1:** Selected bond lengths (Å)

Hg1—Br1	2.4667 (9)
Hg1—Br2	2.4569 (10)
Hg1—N1	2.451 (8)

**Fig. 1.**
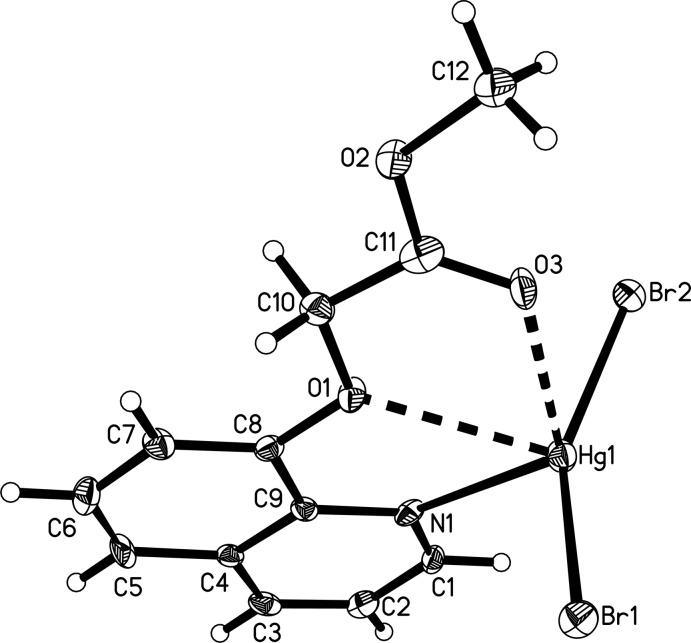
The molecular structure of (I), with 30% probability displacement ellipsoids. The dashed line indicates the weak Hg···O interaction.

**Fig. 2.**
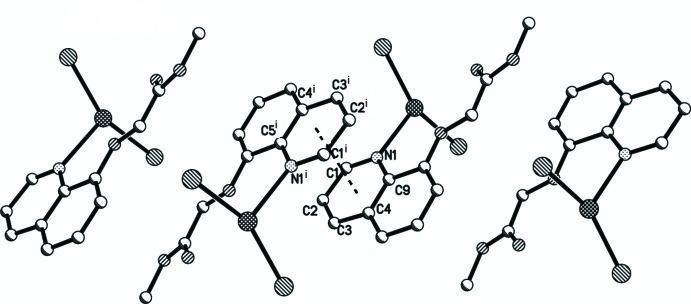
A view of intermolecular π-π interactions, interactions between the parallel quinoline rings of neighbouring complexes [symmetry codes: (i)2 - x, 1 - y, 1 - z].

## supplementary crystallographic information

### Comment

Derivatives of quinoline have received much attention in coordination chemistry (Ghedini *et al.*, 2002; Inomata *et al.*, 1999; Jotterand *et al.*, 2001). 8-quinolinyloxyacetic acid and their derivatives exhibit rich structural variety, and reports of metal complexes with such ligands have increased in recent years (Cheng *et al.*, 2007; Song *et al.*, 2004; Wang, Song *et al.*, 2005; Wang, Fan *et al.*, 2008). In the light of this interest, we have prepared the title Hg^II^ complex with the 8-(methoxycarbonylmethoxy)quinoline ligand, (I).

The title HgBr_2_ adduct, (I), is a mononuclear compound. The Hg^II^ atom exists in a trigonal planar geometry formed by two Br atoms and one quinoline N atom of the 8-(methoxycarbonylmethoxy)quinoline ligand (Fig. 1). The Hg—Br bond lengths are 2.4569 (10) and 2.4667 (9) ° and Hg—N bond length is 2.451 (8) Å. The angles around the Hg atom vary from 100.31 (15) to 152.65 (4) ° (Table 1). There are weak Hg···O interactions with distances of 2.739 (1) Å and 2.905 (1) Å present (Fig. 1). Intermolecular face-to-face π-π interaction stacking is also observed between the parallel quinoline rings of neighbouring complex molecules, with a separation of approximately 3.521 (1) Å (Fig. 2).

### Experimental

Triethylamine (0.0101 g, 0.1 mmol) was added to 8-quinolinyloxyacetic acid (0.0203 g, 0.1 mmol) dissolved in methanol (3 ml). The mixture was stirred for 2 min, Then, the mixture and HgBr_2_ (0.0361 g, 0.1 mmol) were placed in a thick Pyrex tube and heated at 150°C for 3 days. After cooling at a rate of 5 °C per hour to ambient, colorless prism crystals were collected, washed with anhydrous ethanol, and dried at room temperature. The yield is 46% based on 8-quinolinyloxyacetic acid. Analysis found: C, 25.36; H, 1.97; N, 2.42%; calculated for C_12_H_11_Br_2_HgNO_3_: C, 24.95; H, 1.92; N, 2.42%.

### Refinement

H atoms were included in calculated positions and refined as riding, with C—H distances of 0.94 (aromatic), 0.98 (methylene) and 0.97 Å (methyl), and with *U*_iso_(aromatic and methylene) = 1.2*U*_eq_(C) and *U*_iso_(methyl) = 1.5*U*_eq_(C).

### Crystal data

**Table:**

[HgBr_2_(C_12_H_11_NO_3_)]	*Z* = 2
*M**_r_* = 577.63	*F*(000) = 528
Triclinic, *P*1	*D*_x_ = 2.716 Mg m^−^^3^
Hall symbol: -P 1	Mo *K*α radiation, λ = 0.71075 Å
*a* = 7.3132 (8) Å	Cell parameters from 3544 reflections
*b* = 9.9385 (10) Å	θ = 3.2–27.5°
*c* = 10.9902 (10) Å	µ = 16.55 mm^−^^1^
α = 72.102 (11)°	*T* = 223 K
β = 74.966 (12)°	Prism, colorless
γ = 70.740 (11)°	0.50 × 0.40 × 0.20 mm
*V* = 706.40 (14) Å^3^	

### Data collection

**Table:**

Rigaku Saturn diffractometer	2599 independent reflections
Radiation source: fine-focus sealed tube	1949 reflections with *I* > 2σ(*I*)
Graphite monochromator	*R*_int_ = 0.068
Detector resolution: 14.63 pixels mm^-1^	θ_max_ = 25.5°, θ_min_ = 3.2°
ω scans	*h* = −8→8
Absorption correction: multi-scan (*REQAB*; Jacobson, 1998)	*k* = −11→12
*T*_min_ = 0.044, *T*_max_ = 0.137	*l* = −12→13
6021 measured reflections	

### Refinement

**Table:**

Refinement on *F*^2^	Primary atom site location: structure-invariant direct methods
Least-squares matrix: full	Secondary atom site location: difference Fourier map
*R*[*F*^2^ > 2σ(*F*^2^)] = 0.038	Hydrogen site location: inferred from neighbouring sites
*wR*(*F*^2^) = 0.074	H-atom parameters constrained
*S* = 0.80	*w* = 1/[σ^2^(*F*_o_^2^) + (0.0232*P*)^2^] where *P* = (*F*_o_^2^ + 2*F*_c_^2^)/3
2599 reflections	(Δ/σ)_max_ < 0.001
174 parameters	Δρ_max_ = 2.41 e Å^−^^3^
0 restraints	Δρ_min_ = −2.04 e Å^−^^3^

### Special details

**Table:**

Geometry. All e.s.d.'s (except the e.s.d. in the dihedral angle between two l.s. planes) are estimated using the full covariance matrix. The cell e.s.d.'s are taken into account individually in the estimation of e.s.d.'s in distances, angles and torsion angles; correlations between e.s.d.'s in cell parameters are only used when they are defined by crystal symmetry. An approximate (isotropic) treatment of cell e.s.d.'s is used for estimating e.s.d.'s involving l.s. planes.
Refinement. Refinement of *F*^2^ against ALL reflections. The weighted *R*-factor *wR* and goodness of fit *S* are based on *F*^2^, conventional *R*-factors *R* are based on *F*, with *F* set to zero for negative *F*^2^. The threshold expression of *F*^2^ > σ(*F*^2^) is used only for calculating *R*-factors(gt) *etc*. and is not relevant to the choice of reflections for refinement. *R*-factors based on *F*^2^ are statistically about twice as large as those based on *F*, and *R*- factors based on ALL data will be even larger.

### Fractional atomic coordinates and isotropic or equivalent isotropic displacement parameters (Å^2^)

**Table:**

	*x*	*y*	*z*	*U*_iso_*/*U*_eq_	
Hg1	1.01854 (5)	0.82765 (4)	0.67755 (3)	0.02656 (13)	
Br1	1.28595 (13)	0.77673 (12)	0.79728 (8)	0.0324 (3)	
Br2	0.72668 (13)	0.98864 (11)	0.58446 (8)	0.0316 (3)	
O1	0.9081 (8)	0.5789 (7)	0.8222 (5)	0.0260 (15)	
O2	0.5235 (8)	0.7032 (7)	1.0669 (5)	0.0303 (16)	
O3	0.7280 (9)	0.8198 (7)	0.9145 (6)	0.0311 (16)	
N1	1.1332 (9)	0.6134 (8)	0.5841 (6)	0.0211 (17)	
C1	1.2348 (12)	0.6300 (10)	0.4657 (8)	0.023 (2)	
H1	1.2428	0.7252	0.4193	0.028*	
C2	1.3332 (12)	0.5136 (11)	0.4032 (8)	0.029 (2)	
H2	1.4003	0.5321	0.3170	0.035*	
C3	1.3281 (12)	0.3746 (11)	0.4709 (8)	0.026 (2)	
H3	1.3952	0.2947	0.4330	0.032*	
C4	1.2191 (11)	0.3517 (10)	0.6005 (7)	0.020 (2)	
C5	1.2060 (12)	0.2104 (10)	0.6737 (9)	0.029 (2)	
H5	1.2722	0.1280	0.6392	0.035*	
C6	1.0952 (13)	0.1947 (11)	0.7963 (9)	0.033 (3)	
H6	1.0871	0.1005	0.8461	0.040*	
C7	0.9956 (12)	0.3148 (11)	0.8475 (8)	0.026 (2)	
H7	0.9182	0.3007	0.9309	0.031*	
C8	1.0058 (11)	0.4532 (10)	0.7809 (8)	0.021 (2)	
C9	1.1234 (11)	0.4738 (10)	0.6524 (8)	0.019 (2)	
C10	0.7609 (12)	0.5656 (11)	0.9358 (8)	0.027 (2)	
H10A	0.8196	0.4930	1.0083	0.033*	
H10B	0.6588	0.5320	0.9205	0.033*	
C11	0.6734 (13)	0.7084 (12)	0.9681 (8)	0.031 (3)	
C12	0.4182 (13)	0.8376 (12)	1.1065 (9)	0.041 (3)	
H12A	0.5058	0.8692	1.1378	0.062*	
H12B	0.3083	0.8212	1.1754	0.062*	
H12C	0.3696	0.9130	1.0331	0.062*	

### Atomic displacement parameters (Å^2^)

**Table:**

	*U*^11^	*U*^22^	*U*^33^	*U*^12^	*U*^13^	*U*^23^
Hg1	0.0287 (2)	0.0224 (2)	0.02744 (19)	−0.00666 (15)	−0.00615 (14)	−0.00383 (15)
Br1	0.0335 (5)	0.0360 (7)	0.0283 (5)	−0.0116 (5)	−0.0083 (4)	−0.0042 (4)
Br2	0.0288 (5)	0.0271 (6)	0.0362 (5)	−0.0052 (4)	−0.0077 (4)	−0.0051 (4)
O1	0.031 (3)	0.021 (4)	0.022 (3)	−0.012 (3)	0.010 (3)	−0.008 (3)
O2	0.036 (3)	0.029 (4)	0.023 (3)	−0.013 (3)	0.014 (3)	−0.014 (3)
O3	0.040 (4)	0.017 (4)	0.032 (3)	−0.009 (3)	0.005 (3)	−0.008 (3)
N1	0.020 (4)	0.028 (5)	0.018 (4)	−0.012 (3)	0.003 (3)	−0.008 (3)
C1	0.027 (5)	0.019 (6)	0.028 (5)	−0.010 (4)	−0.012 (4)	−0.001 (4)
C2	0.025 (5)	0.036 (7)	0.028 (5)	−0.013 (5)	−0.002 (4)	−0.007 (5)
C3	0.019 (4)	0.031 (6)	0.034 (5)	0.000 (4)	−0.009 (4)	−0.018 (5)
C4	0.014 (4)	0.021 (6)	0.022 (4)	−0.002 (4)	−0.001 (3)	−0.005 (4)
C5	0.033 (5)	0.014 (6)	0.043 (5)	0.000 (4)	−0.010 (4)	−0.014 (4)
C6	0.040 (6)	0.023 (6)	0.033 (5)	−0.014 (5)	−0.005 (4)	0.002 (5)
C7	0.024 (5)	0.025 (6)	0.025 (5)	−0.002 (4)	−0.001 (4)	−0.010 (4)
C8	0.018 (4)	0.021 (6)	0.026 (4)	−0.006 (4)	−0.003 (3)	−0.010 (4)
C9	0.016 (4)	0.019 (6)	0.024 (4)	−0.003 (4)	−0.006 (3)	−0.007 (4)
C10	0.025 (5)	0.028 (6)	0.027 (5)	−0.006 (4)	0.000 (4)	−0.009 (4)
C11	0.029 (5)	0.046 (8)	0.017 (4)	−0.013 (5)	−0.007 (4)	0.000 (5)
C12	0.034 (5)	0.038 (8)	0.047 (6)	−0.012 (5)	0.009 (5)	−0.015 (5)

### Geometric parameters (Å, º)

**Table:**

Hg1—Br1	2.4667 (9)	C4—C9	1.396 (13)
Hg1—Br2	2.4569 (10)	C4—C5	1.409 (12)
Hg1—N1	2.451 (8)	C5—C6	1.372 (12)
O1—C8	1.367 (11)	C5—H5	0.9400
O1—C10	1.425 (9)	C6—C7	1.378 (14)
O2—C11	1.330 (10)	C6—H6	0.9400
O2—C12	1.440 (12)	C7—C8	1.362 (12)
O3—C11	1.225 (11)	C7—H7	0.9400
N1—C1	1.312 (10)	C8—C9	1.444 (11)
N1—C9	1.375 (11)	C10—C11	1.463 (14)
C1—C2	1.418 (14)	C10—H10A	0.9800
C1—H1	0.9400	C10—H10B	0.9800
C2—C3	1.361 (12)	C12—H12A	0.9700
C2—H2	0.9400	C12—H12B	0.9700
C3—C4	1.431 (11)	C12—H12C	0.9700
C3—H3	0.9400		
			
N1—Hg1—Br2	106.34 (15)	C7—C6—H6	119.5
N1—Hg1—Br1	100.31 (15)	C8—C7—C6	122.0 (8)
Br2—Hg1—Br1	152.65 (4)	C8—C7—H7	119.0
C8—O1—C10	116.2 (7)	C6—C7—H7	119.0
C11—O2—C12	117.3 (8)	C7—C8—O1	126.1 (8)
C1—N1—C9	117.8 (8)	C7—C8—C9	118.6 (9)
C1—N1—Hg1	116.8 (6)	O1—C8—C9	115.3 (7)
C9—N1—Hg1	124.7 (5)	N1—C9—C4	122.2 (7)
N1—C1—C2	124.2 (8)	N1—C9—C8	119.0 (8)
N1—C1—H1	117.9	C4—C9—C8	118.8 (8)
C2—C1—H1	117.9	O1—C10—C11	109.4 (7)
C3—C2—C1	118.5 (9)	O1—C10—H10A	109.8
C3—C2—H2	120.8	C11—C10—H10A	109.8
C1—C2—H2	120.8	O1—C10—H10B	109.8
C2—C3—C4	119.0 (10)	C11—C10—H10B	109.8
C2—C3—H3	120.5	H10A—C10—H10B	108.2
C4—C3—H3	120.5	O3—C11—O2	122.8 (10)
C9—C4—C5	120.5 (8)	O3—C11—C10	126.2 (8)
C9—C4—C3	118.2 (8)	O2—C11—C10	111.0 (8)
C5—C4—C3	121.3 (9)	O2—C12—H12A	109.5
C6—C5—C4	119.1 (9)	O2—C12—H12B	109.5
C6—C5—H5	120.5	H12A—C12—H12B	109.5
C4—C5—H5	120.5	O2—C12—H12C	109.5
C5—C6—C7	121.0 (9)	H12A—C12—H12C	109.5
C5—C6—H6	119.5	H12B—C12—H12C	109.5
			
Br2—Hg1—N1—C1	79.0 (6)	C1—N1—C9—C4	0.9 (11)
Br1—Hg1—N1—C1	−94.8 (6)	Hg1—N1—C9—C4	−169.3 (6)
Br2—Hg1—N1—C9	−110.8 (6)	C1—N1—C9—C8	−177.4 (7)
Br1—Hg1—N1—C9	75.4 (6)	Hg1—N1—C9—C8	12.4 (10)
C9—N1—C1—C2	0.7 (12)	C5—C4—C9—N1	179.8 (7)
Hg1—N1—C1—C2	171.6 (6)	C3—C4—C9—N1	−1.0 (11)
N1—C1—C2—C3	−2.1 (13)	C5—C4—C9—C8	−1.9 (12)
C1—C2—C3—C4	1.9 (12)	C3—C4—C9—C8	177.3 (7)
C2—C3—C4—C9	−0.5 (11)	C7—C8—C9—N1	179.5 (7)
C2—C3—C4—C5	178.7 (7)	O1—C8—C9—N1	1.8 (11)
C9—C4—C5—C6	1.0 (12)	C7—C8—C9—C4	1.1 (11)
C3—C4—C5—C6	−178.2 (8)	O1—C8—C9—C4	−176.5 (7)
C4—C5—C6—C7	0.8 (14)	C8—O1—C10—C11	179.0 (7)
C5—C6—C7—C8	−1.6 (14)	C12—O2—C11—O3	2.8 (12)
C6—C7—C8—O1	178.0 (8)	C12—O2—C11—C10	−177.8 (7)
C6—C7—C8—C9	0.6 (13)	O1—C10—C11—O3	−6.2 (12)
C10—O1—C8—C7	−9.9 (12)	O1—C10—C11—O2	174.5 (6)
C10—O1—C8—C9	167.5 (7)		
